# Supplementary material for: Innate and adaptive signals enhance differentiation and expansion of dual-antibody autoreactive B cells in lupus
Source: Nat Commun. 2018 Sep 28;9:3973. doi: 10.1038/s41467-018-06293-z (PMC6162205; doi:10.1038/s41467-018-06293-z)
Supplement: Supplementary file 1 — Supplementary Information [file 41467_2018_6293_MOESM1_ESM.pdf]

Supplementary Material

Innate and adaptive signals enhance differentiation and expansion of dual-antibody autoreactive B cells in lupus

Sang et al.

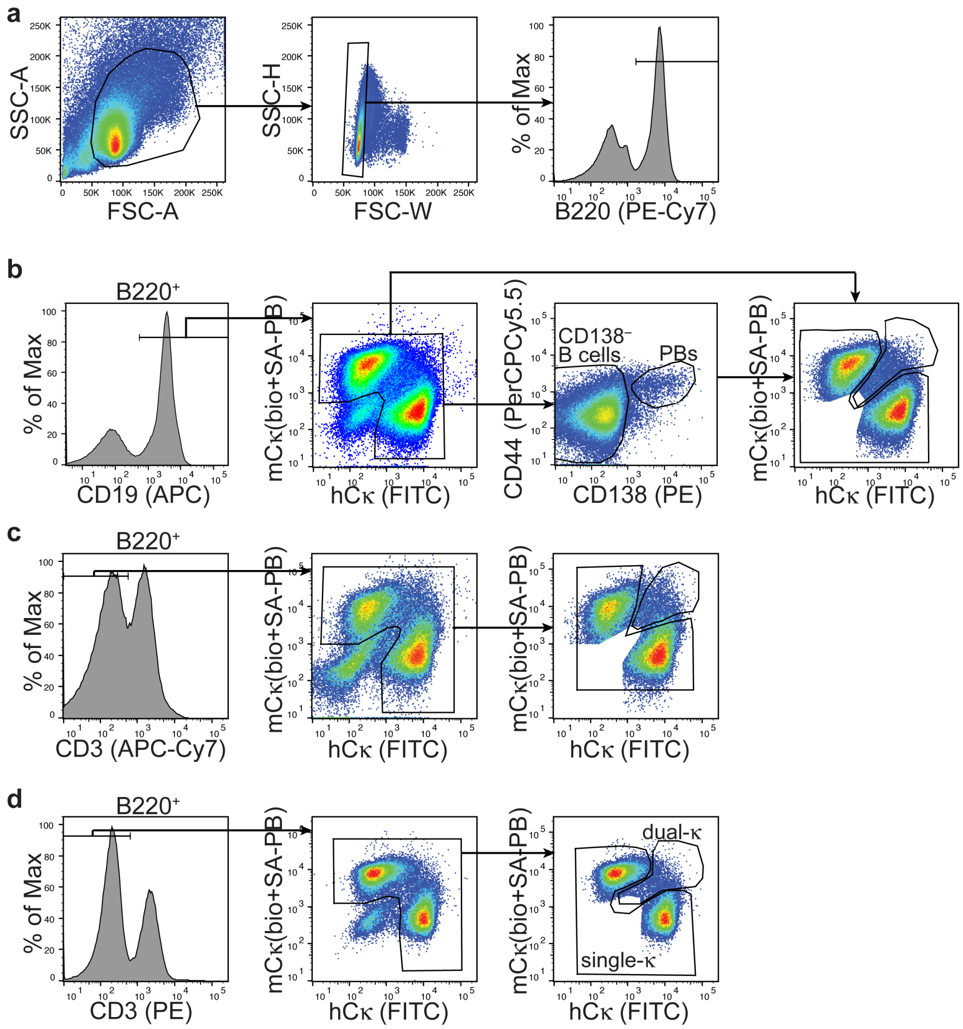

Supplementary Figure 1. *Gating strategies for flow cytometric analyses of cell proliferation and function*. (**a**) Gating of B220$^+$ single lymphocytes that preceded the gating strategies described in panels (b-d). (**b**) B220$^+$ single lymphocytes gated as in panel (a) were gated on CD19$^+$ and then Igκ$^+$ cells to analyze the expression of MHCII (Fig. 4a,b), IL-21R (Fig. 4e-g), PD-L1 (Fig. 4h), and the frequency of germinal center B cells (Fig. 4j,k) in single and dual-κ populations. Alternatively, CD19$^+$Igκ$^+$ cells were further gated to distinguish CD138$^+$ plasmablasts (PBs) and CD138$^-$ B cells that were then electronically separated into single and dual-κ cells that were analyzed for the expression of Ki67 (Fig. 1a,b) and MHCII (Fig. 4c, for PBs only). (**c**) B220$^+$ single lymphocytes gated (from panel a) were gated to remove CD3 cells and then to discriminate single and dual-κ cells that were analyzed for EdU incorporation as displayed in Figs. 1c,d and 5f. (**d**) B220$^+$ single lymphocytes (from panel a) were gated to remove CD3 cells and then to discriminate single and dual-κ cells that were analyzed for c-Myc expression as displayed in Fig. 1e,f.

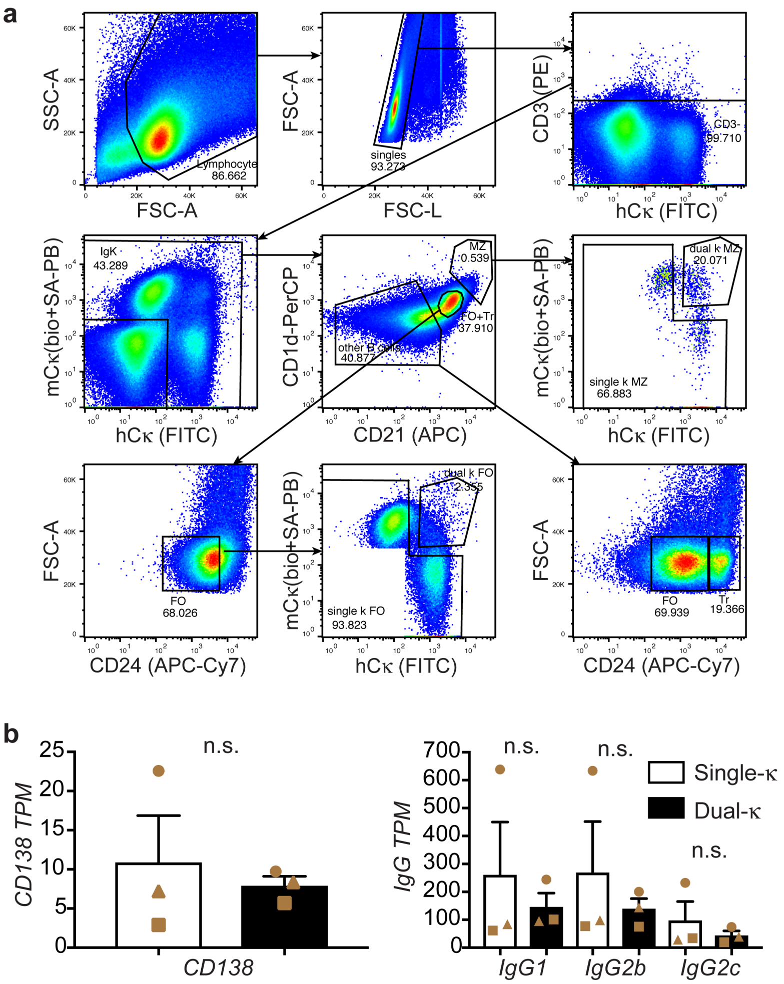

Supplementary Figure 2. *Gating strategy for sorting cells used for RNAseq analyses*. (**a**) Gating strategy to sort MRL/*lpr-Igk*$^{m/h}$ single and dual-κ splenic follicular (FO) and marginal zone (MZ) B cells used for the RNAseq analyses presented in Figs. 2, 3a, 4d. Before staining and sorting, cells were depleted of CD3$^+$ T cells by complement-mediated lysis. Any remaining CD3$^+$ cells were removed by gating as shown (upper right plot). FO B cells were identified by intermediate expression of CD21 and CD1d (center plot) and low CD24 abundance (bottom left plot), and this latter was based on the analysis of the B cell population that included transitional (Tr) B cells (bottom right plot). MZ B cells were identified by very high expression of CD21 and CD1d (center plot). (**b**) *CD138* (left) and *IgG* (right) RNA transcript counts in single or dual-κ FO and MZ B cells sorted as shown in (a) from the spleen of 16 wk MRL/*lpr-Igk*$^{m/h}$ mice. Data are shown as mean transcripts per million (TPM) ± SEM. Data were combined from three independent biological replicates analyzed in one experiment. n.s., not significant. Significance was assessed by Student's *t* or Mann-Whitney tests.

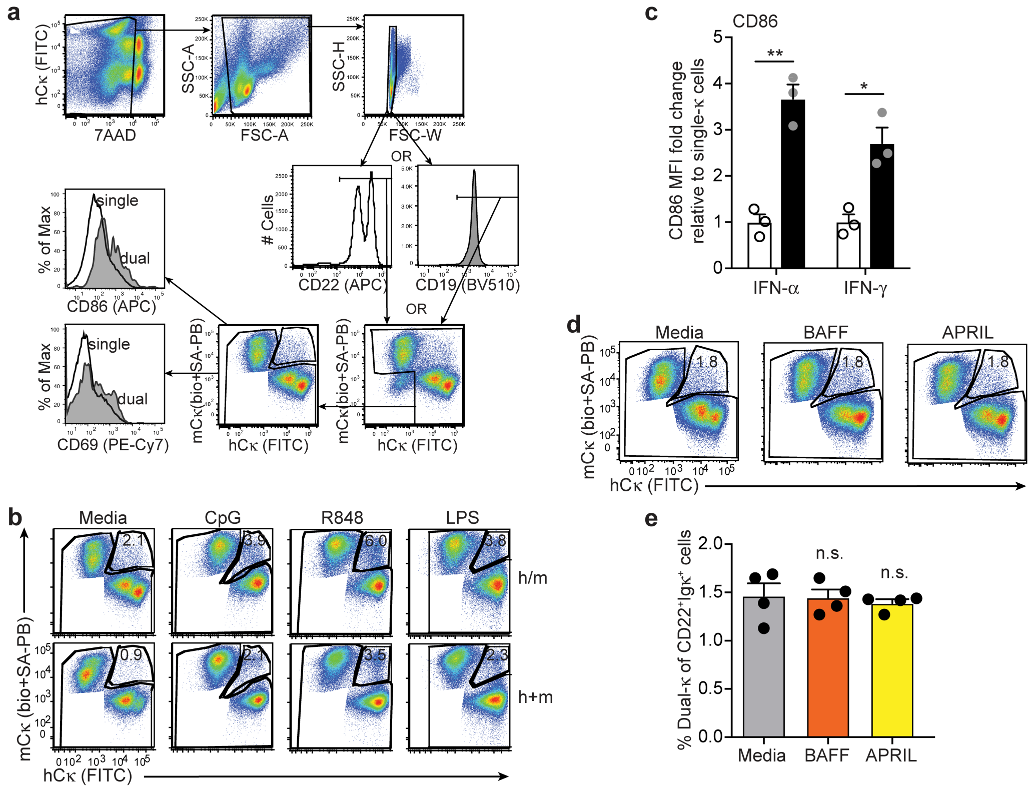

Supplementary Figure 3. *In vitro responses of single and dual-κ B cell to TLR agonists, IFNs, and B cell survival factors*. (**a**) Gating strategy for the analyses of purified B cells cultured with TLR agonists, IFNs and survival factors. Live single cells, gated as shown on top row, were then gated for either CD22$^+$ cells (data in Figs. 3b,c and panels b,d,e in here) or CD19$^+$ cells (data in Figs. 3d,e,f,g and panel c in here) that were then discriminated for single and dual-κ cells. These were either enumerated or analyzed for the expression of activation markers. (**b-e**) Spleen B cells, isolated by negative selection of CD43$^+$ cells, were cultured for 24-48 h with indicated stimuli. (**b**) Representative analysis of B cells cultured with TLR agonists. The plots depict the gating of single and dual-κ cells in B cell cultures from MRL/*lpr-Igk*$^{m/h}$ (top row) or a mix of MRL/*lpr-Igk*$^{h/h}$ and MRL/*lpr-Igk*$^{m/m}$ (h+m) (bottom row) spleen cells. The cells were first gated as 7AAD$^-$CD22$^+$Igκ$^+$ as shown in panel (a). For each experimental condition, the frequency of non-specific dual-κ B cell events measured in h+m cultures was subtracted from the frequency of dual-κ B cells in MRL/*lpr-Igk*$^{m/h}$ B cell cultures. (**c**) MFI ± SEM of CD86 in MRL/*lpr-Igk*$^{m/h}$ dual-κ B cells, as a fold change relative to levels in single-κ cells, after 24h of culture with IFNα or IFNγ. Single and dual-κ cells were analyzed as shown in panel (a) via CD19$^+$ cell gating. Data are from one experiment using 12 wk old MRL/*lpr-Igk*$^{m/h}$ mice (N=3). (**d**) Representative plots showing the frequencies of dual-κ splenic B cells from MRL/*lpr-Igk*$^{m/h}$ mice following 48h culture with BAFF or APRIL. Cells were analyzed as shown in panel (a) via CD22$^+$ cell gating. (**e**) Mean frequency ± SEM of dual-κ B cells in cultures with BAFF or APRIL described in panel (d). N=4, from one experiment. *P < 0.05, **P < 0.01, n.s., not significant. Significance was assessed by Student's *t* or Mann-Whitney tests. Symbols in bar graphs represent individual mice.

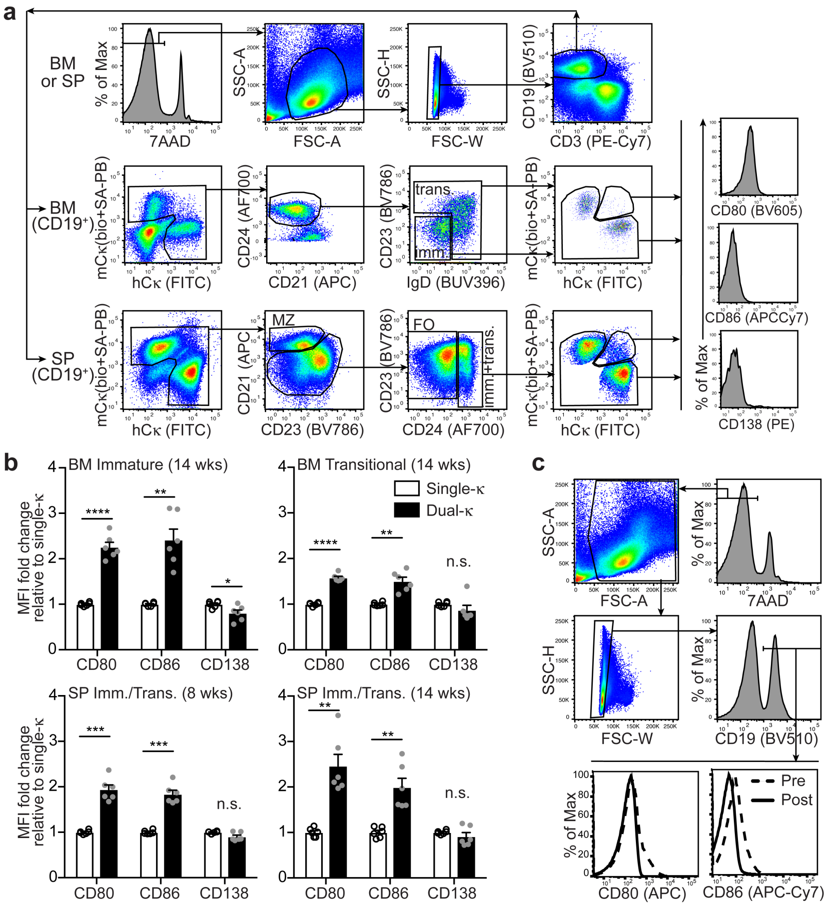

Supplementary Figure 4. *Expression of activation markers in immature, transitional and naïve B cells*. **(a)** Gating strategy for the analysis shown in panel (b) of CD80, CD86, and CD138 expression by bone marrow (BM) and spleen (SP) immature and transitional B cells of MRL/*lpr-Igk*$^{m/h}$ mice. Part of this strategy was also used for the analyses of data shown in Fig. 5c (frequency of dual-κ in spleen immature+transitional cells) and Supplementary Fig. 6c (expression of activation marker by spleen FO and MZ single and dual-κ B cells). (**b**) CD80, CD86, and CD138 MFI levels ± SEM on CD3^{-} CD19^{+} dual-κ cells relative to single-κ cells (fold-change) in the following populations: BM Igκ^{+}CD24^{high}CD23^{-}IgD^{-} immature B cells, BM Igκ^{+}CD24^{high}CD23$^{-/+}$IgD^{+} transitional B cells, and SP Igκ^{+}CD21$^{-/+}$CD24^{high} immature/transitional B cells, gated as shown in panel (a). Data were combined from two independent experiments using 14 wk old MRL/*lpr-Igk*$^{m/h}$ mice (N=6 total). Symbols represent individual mice. Similar results were obtained from groups of 8 wk old mice (N=6) as shown for spleen cells. (**c**) Analysis of naïve B cells isolated by magnetic depletion of CD43^{+}CD80^{+}CD86^{+} cells from the spleen of 14 wk old MRL/*lpr-Igk*$^{m/h}$ mice (representative of N=4) to verify the absence of activated cells. CD19^{+} cells from samples before (dashed line) and after (solid line) magnetic cell depletion were gated as shown on top two rows and then analyzed for the expression of CD80 and CD86. Data are from one experiment. *P < 0.05, **P < 0.01, ***P < 0.001, ****P < 0.0001; n.s., not significant. Significance was assessed by Student's *t* or Mann-Whitney tests.

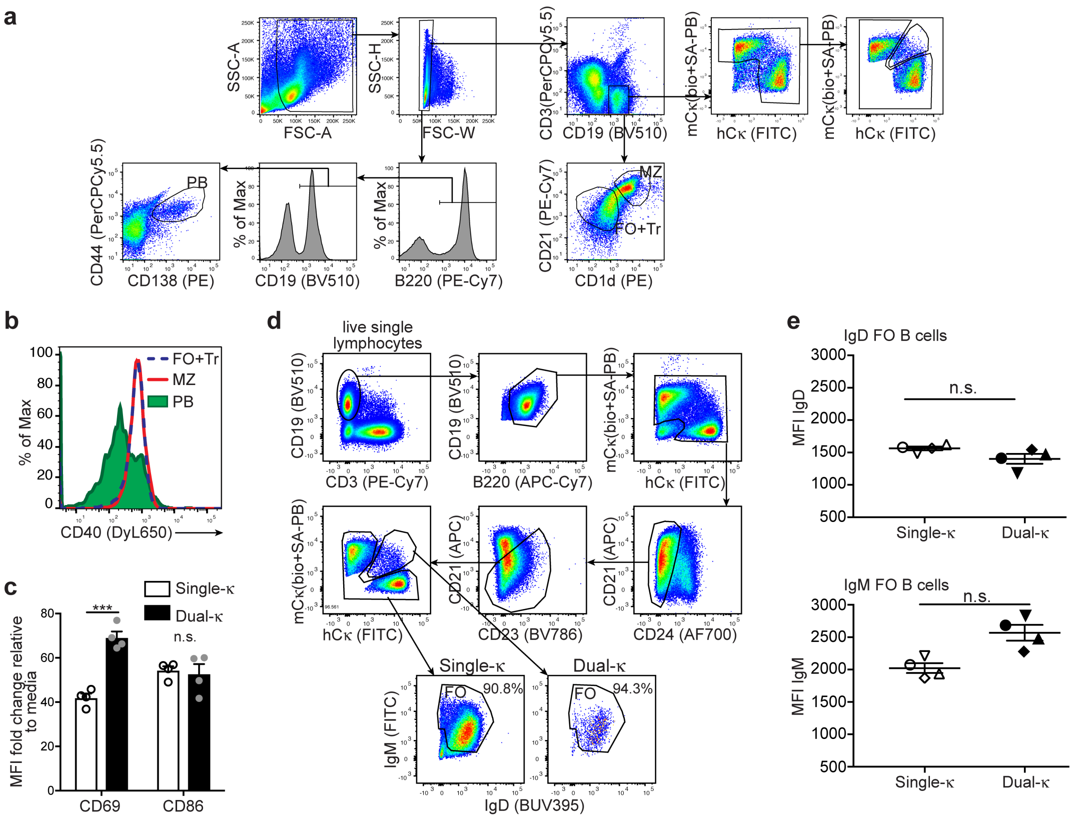

Supplementary Figure 5. *Analysis of CD40, IgD and IgM expression*. (**a**) Gating strategy for the analysis of CD40 expression on follicular&transitional (FO+Tr) B cells, marginal zone (MZ) B cells, and plasmablasts (PBs) displayed in panel (b). Part of this gating strategy was also employed for the analysis of CD80, CD86, and CD40 expression on $CD3^{-}CD19^{+}$ single and dual-κ B cells displayed in Fig. 4h. (**b**) Representative flow cytometric analysis (from two independent experiments, N=5 total) of CD40 expression on FO+Tr B cells (dashed blue line), MZ B cells (solid red line), and PB (green) gated as in panel (a). (**c**) Bar graph of relative CD69 and CD86 MFI in untouched ($CD43^{-}$) B cells cultured for 48 h with anti-CD40 antibodies. Flow cytometric analysis of these cells was done as described in Supplementary Fig. 3a. Data for both single and dual-κ B cells (from one experiment using 14 wk MRL/*lpr-Igk*$^{m/h}$ mice, N=4) are shown as a mean MFI fold change ± SEM relative to corresponding B cells cultured in media without stimuli. Symbols represent individual mice. (**d**) Representative flow plots showing the gating strategy for the analysis of IgM and IgD expression by MRL/*lpr-Igk*$^{m/h}$ FO single-κ and dual-κ splenic B cells. Cells were first gated on live single lymphocytes as shown in Supplementary Fig. 4a, top row. These cells were then gated as $CD3^{-}CD19^{+}$ cells following by gating $B220^{+}$ and $Ig\kappa^{+}$ cells. These latter were gated on $CD24^{low}$ cells followed by gating $CD23^{+}CD21^{+}$ cells and excluding $CD21^{high}$ MZB cells. (**e**) IgD and IgM protein levels on FO B cells gated as in (d). The graphs show MFI ± SEM of IgD (top) and IgM (bottom) on single-κ (open symbols) and dual-κ (closed symbols) B cells from N=4 MRL/*lpr-Igk*$^{m/h}$ mice in one experiment. Symbols of similar shape represent cells from the same mouse. ***P < 0.001. n.s., not significant. Significance was assessed by Student's *t* or Mann-Whitney tests.

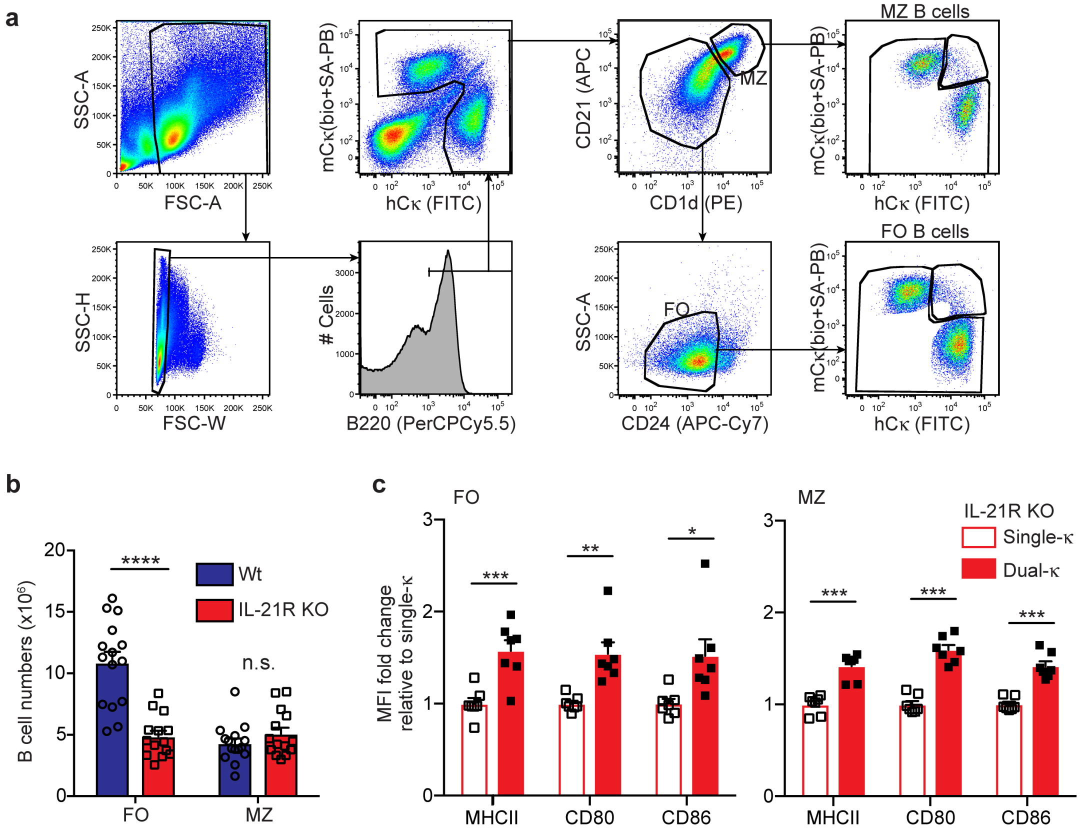

Supplementary Figure 6. *Contribution of IL-21 to follicular and marginal zone B cells of MRL/lpr mice*. (**a**) Representative gating strategy of CD21^{+}CD1d^{low}CD24^{low} FO B cells and CD21^{high}CD1d^{high} MZ B cells within the B220^{+}Igκ^{+} spleen cell populations of wt and IL-21R KO MRL/*lpr-Igk*$^{m/h}$ mice for the data analyses shown in panel (b) and Figs. 4c and 5a,b. (**b**) Mean absolute numbers ± SEM of FO and MZ B cells in the spleen of wt (blue bars) or IL-21R KO (red bars) MRL/*lpr-Igk*$^{m/h}$ mice gated as in (a). Data are combined from five independent experiments with N=15 wt and 14 KO 15-20 wk old mice total. (**c**) MHCII, CD80, and CD86 MFI expression levels ± SEM on CD3^{-}CD19^{+} single-κ or dual-κ cells gated as CD21^{low}CD23^{+}CD24^{low} FO (left) and CD21^{high}CD23^{-} MZ (right) B cells, as depicted in Supplementary Fig. 4a, SP. Data, shown as a fold change relative to levels on single-κ cells, were combined from two independent experiments using (N=7) 10-17 wk old IL-21R KO MRL/*lpr-Igk*$^{m/h}$ mice. *P < 0.05, **P < 0.01, ***P < 0.001, ****P < 0.0001; n.s., not significant. Significance was assessed by Student's *t* or Mann-Whitney tests. Symbols in bar graphs represent individual mice.

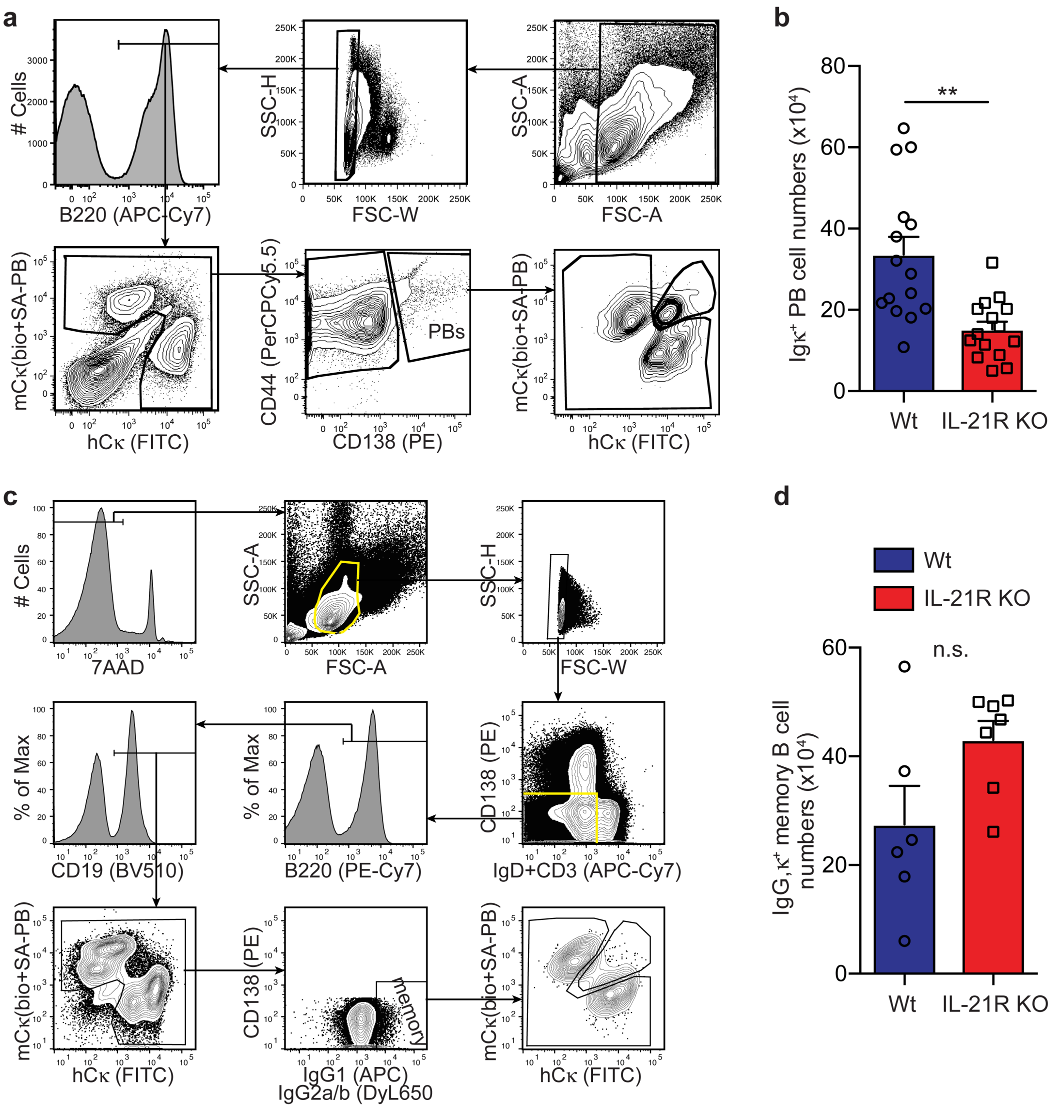

Supplementary Figure 7. *Contribution of IL-21 to plasmablast and memory B cells of MRL/lpr mice*. (**a**) Gating strategy for the analysis of plasmablasts (PBs) presented in panel (b) and Fig. 5d,e. PBs were identified by high expression of CD44 and CD138 (center bottom plot). (**b**) Total numbers of PBs ± SEM cells in spleen of wt and IL-21R KO MRL/*lpr-Igk$^{m/h}$* 15-20 wk old mice, analyzed as described in panel (a). Data are combined from five independent experiments with N=15 mice per group. (**c**) Gating strategy for the analysis of memory B cells presented in panel (d) and Fig. 5g,h. Memory Ig class switched B cells were identified as IgD$^-$CD138$^-$ and the expression of IgG1/2a/b. (**d**) Total numbers of memory IgG$^+$ B cells ± SEM cells in the spleens of wt and IL-21R KO MRL/*lpr-Igk$^{m/h}$* mice gated as described in panel (c). Data are combined from three independent experiments with N=7 wt and 6 KO mice total. **P < 0.01; n.s., not significant. Significance was assessed by Student's *t* or Mann-Whitney tests. Symbols in bar graphs represent individual mice.

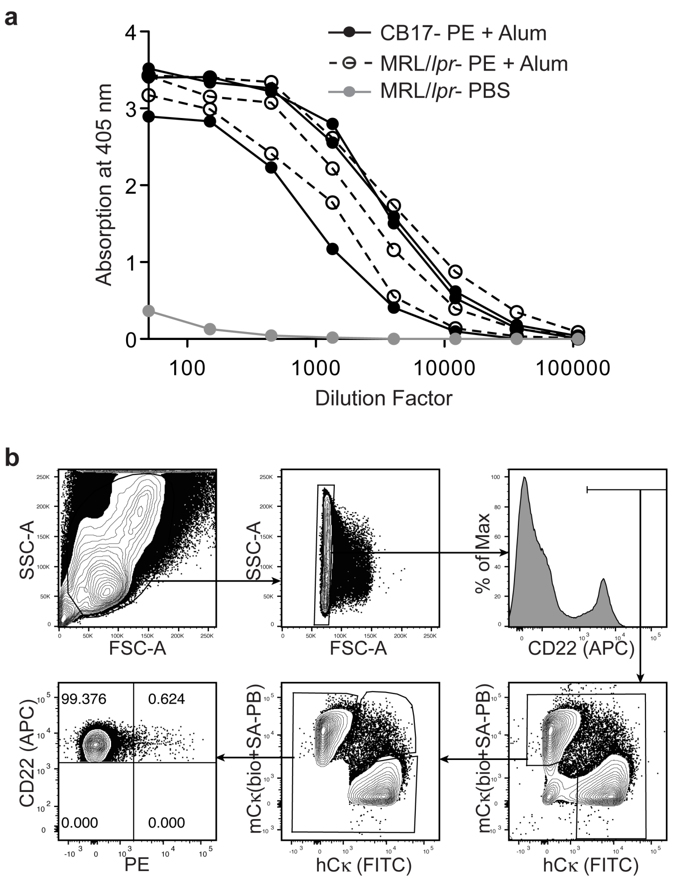

Supplementary Figure 8. *Analysis of anti-PE antibodies and PE-reactive B cells*. (**a**) Anti-PE IgG antibodies measured by ELISA in the sera of CB17-*Igk*$^{m/h}$ mice (solid black line, filled circles) and 6-7 wk old MRL/*lpr-Igk*$^{m/h}$ mice (dotted black line, open circles), 7 d after immunization with PE+Alum. Data from three immunized mice per strain in one experiment are shown. Serum from one MRL/*lpr-Igk*$^{m/h}$ mouse injected with PBS (solid gray line, filled circles) is shown as a negative control. (**b**) Gating strategy for the analyses of B cells binding PE shown in Fig. 6.
